# Molecular Characterization of the Iron-Containing Alcohol Dehydrogenase from the Extremely Thermophilic Bacterium *Pseudothermotoga hypogea*

**DOI:** 10.3390/microorganisms12020311

**Published:** 2024-02-01

**Authors:** Liangliang Hao, Zainab Ayinla, Kesen Ma

**Affiliations:** Department of Biology, University of Waterloo, Waterloo, ON N2L 3G1, Canadazayinla@uwaterloo.ca (Z.A.)

**Keywords:** hyperthermophilic bacteria, *Pseudothermotoga hypogea*, iron-containing alcohol dehydrogenase, alcohol fermentation, enzymology

## Abstract

*Pseudothermotoga hypogea* is an extremely thermophilic bacterium capable of growing at 90 °C and producing ethanol, which is catalyzed by an alcohol dehydrogenase (ADH). The gene encoding *P. hypogea* ADH (*Ph*ADH) was cloned, sequenced and over-expressed. The gene sequence (1164 bp) was obtained by sequencing all fragments of the gene, which were amplified from the genomic DNA. The deduced amino acid sequence showed high identity to iron-containing ADHs from other *Thermotoga* species and harbored typical iron- and NADP-binding motifs, Asp195His199His268His282 and Gly39Gly40Gly41Ser42, respectively. Structural modeling showed that the N-terminal domain of *Ph*ADH contains an α/β-dinucleotide-binding motif and that its C-terminal domain is an α-helix-rich region containing the iron-binding motif. The recombinant *Ph*ADH was soluble, active, and thermostable, with a subunit size of 43 ± 1 kDa revealed by SDS-PAGE analyses. The recombinant *Ph*ADH (69 ± 2 U/mg) was shown to have similar properties to the native enzyme. The optimal pH values for alcohol oxidation and aldehyde reduction were 11.0 and 8.0, respectively. It was also thermostable, with a half-life of 5 h at 70 °C. The successful expression of the recombinant *Ph*ADH in *E. coli* significantly enhanced the yield of enzyme production and thus will facilitate further investigation of the catalytic mechanisms of iron-containing ADHs.

## 1. Introduction

Alcohol dehydrogenases (ADHs), belonging to the oxidoreductase class of enzymes, catalyze the production of alcohols and their oxidation to the corresponding aldehydes or ketones [1,2]. They are ubiquitous in all three domains of life and are classified into three groups based on their sizes: short-chain (group I), medium-chain (group II), and long-chain (group III) ADHs, respectively. Based on the metal content, ADHs can be classified into the following categories: metal-free ADHs, zinc-containing ADHs, and Fe-dependent ADHs, which correspond to group I, II, and III ADHs, respectively [3,4]. Zinc ions have catalytic or structural functions in several enzymes including hyperthermophilic zinc-containing ADHs; however, only a limited number of iron-dependent ADHs are characterized, owing to their instability [5]. Some of them have been over-expressed in the mesophilic heterologous *E. coli* system, and a detailed 3-D structure was resolved [6]. Iron-containing ADHs are found to be present primarily in hyperthermophiles [5].

Hyperthermophiles are classified as members of the domains Archaea and Bacteria [7,8]. Many of them produce alcohols, such as ethanol, as metabolic end products. Approximately ten hyperthermophilic Fe-containing ADHs have been characterized from: *Thermococcus litoralis* [9], *Thermococcus paralvinellae* strain ES-1 [10,11], *Thermococcus zilligii* strain AN1 [12], *Thermococcus hydrothermalis* [13], *Thermococcus barophilus Ch5* (Tba ADH 547) [14], *Thermococcus barophilus Ch5* (Tba ADH 641) [15], *Pyrococcus horikoshii* OT3 [16], *Pseudothermotoga hypogea* [17], *Thermotoga neapolitana* [18], and *Hyperthermus butylicus* (ADH2) [19]. They catalyze the reversible aldehyde reduction and alcohol oxidation, and the optimal pH values for reduction are within the range of 6–8, while the values for oxidation fall within a range of 8–11. Values of *K*m for the oxidation (10–92 mM) are much higher than those for reduction (0.1–35 mM), while the catalytic efficiency of the reduction is much higher than that of oxidation, indicating that their physiological function is to catalyze the production of alcohol at high temperatures [5,18].

The majority of the known iron-containing ADHs are from hyperthermophilic archaea. However, only the iron-containing ADHs from *T. paralvinellae* strain ES-1 [11], *T. hydrothermalis* [13], *T*. *barophilus Ch5* (Tba ADH 547) [14], *T. barophilus Ch5* (Tba ADH 641) [15], and NADP-dependent iron-containing 1,3-propanediol dehydrogenase encoded by the TM0920 gene of *Thermotoga maritima* were reported to be successfully expressed in *E. coli* [6]. The three-dimensional (3-D) structure of the *T. maritima* ADH that has been solved at 1.30 Å resolution is the only available detailed structure of the iron-containing ADH from hyperthermophiles [6].

*Pseudothermotoga hypogea* is an anaerobic, extremely thermophilic bacterium. It belongs to the order *Thermotogales*, which comprise a group of rod-shaped, strictly anaerobic bacterium which have a peculiar outer sheath-like structure around the cell known as a ‘toga’ [20,21]. Many of the *Thermotogales* are hyperthermophiles that can grow optimally at temperatures of 80 °C or above [22,23] with some being capable of growing at 90 °C [23,24]. Of great interest to biotechnological applications are hyperthermophiles, that mainly grow at such high temperatures [25,26,27] because fermentation at high temperatures lessens the pretreatment requirements and contaminations [28,29]. Although *P. hypogea* grows at 55–90 °C, its optimum growth temperature is 70 °C at a pH of 7.0. It utilizes a broad range of substrates including glucose, xylose and xylan as carbon and energy sources and produces ethanol, acetate, CO_2_ and hydrogen [20]. ADH is considered to be a key enzyme responsible for alcohol fermentation in *P. hypogea*, which catalyzes the interconversion between alcohols and the corresponding aldehydes. The *P. hypogea* ADH (*Ph*ADH) is the first iron-containing ADH purified from the hyperthermophilic bacteria [17]. The recombinant *Ph*ADH that is expressed and purified from *E. coli* could replace the enzymes from the native sources, and the higher level of ADHs produced in *E. coli* will facilitate the preparation of protein crystals for the determination of the three-dimensional structure of this distinct class of ADHs. In this work, we report on the cloning and overexpression in *E. coli* of ADH from *P. hypogea*, and the purification and characterization of the recombinant enzyme. Furthermore, the recombinant enzyme properties were compared with those of the native enzyme [17]. High yields of recombinant ADH will be beneficial for further studies on the enzyme.

## 2. Materials and Methods

### 2.1. Growth of P. hypogea

*P. hypogea* (DSM 11164) was obtained from the Deutsche Sammlung von Mikroorganismen und Zellkulturen, Germany, and it was grown as described previously [17,20]. All chemicals used were commercially available, and they were purchased from Sigma-Aldrich Canada Ltd. (Oakville, ON, Canada) and Fischer Scientific (Ottawa, ON, Canada). One liter of the medium contained 1 g of NH_4_Cl, 0.3 g of K_2_HPO_4_, 0.3 g of KH_2_PO_4_, 0.2 g of MgCl_2_·6H_2_O, 0.1 g of CaCl_2_·2H_2_O, 0.1 g of KCl, 2.0 g of yeast extract, 2.0 g of trypticase, 10 mL of trace mineral element solution, and 0.05 mg of resazurin. The medium was sealed in serum bottles, autoclaved, and degassed; the gas phase was refilled using pure N_2_. Before inoculation, 1 mL of 25% Na_2_S_2_O_3_, 0.14 mL 15% cysteine and 0.4 mL 3% Na_2_S were added to 50 mL of the medium.

### 2.2. DNA Sequencing and Sequence Analysis

DNA fragments carrying the target gene were amplified from genomic DNA directly by using PCR. Based on the previously identified N-terminal (MENFVFHNPTKLIFG) and internal sequence (LMLYGGGSI) within the native *Ph*ADH [17], two primer sequences (forward 5′-A TGGAGAACTTCGTCTTCCACAATCC-3′; reverse 5′ TATCGATCCACCACCGTATAGCATCAG-3′) were synthesized and used for PCR reaction with parameters set up at an initial denaturation at 95 °C for 2 min; 36 cycles were followed with denaturation at 95 °C for 20 s; annealing at 55 °C for 20 s; extension at 70 °C for 30 s; and a final extension at 70 °C for 3 min. The ~150 bp PCR products were separated by agarose gel, purified, and sequenced (Molecular Biology Core Facility, University of Waterloo). The BLAST search of the sequenced fragment indicated its highest similarity to the putative NADH-dependent butanol dehydrogenase from *T. maritima* TM0820. Considering the high-sequence similarity among the *Thermotoga* species, gradual primer walking was performed and the primers were designed based on the homology of genes encoding the *Ph*ADH among *Thermotoga* species, including *Thermotoga maritima, Thermotoga petrophila*, and *Thermotoga* sp. RQ2. The primers that were used included the following: 5′-AACTTCGTCTTCCACAATCC-3′, 5′-TCATCTCCGTTCCTGTCG-3′, 5′-GTGTGTGCTATTGCGTCG-3′, 5′-ACTGAGATGAACGGAAACG-3′, and 5′-GCGACGATAGCCCTGAACA-3′. The final product of the nucleotide sequence cloned from *P. hypogea* genome was 1164 bp, encoding 387 amino acid residues (GenBank: AMT84600.1).

### 2.3. Expression of Recombinant PhADH

The *Ph*ADH gene was inserted into vector pET-30a (5360 bp, PT7, Kan; Novagen, WI, USA) and over-expressed in *E. coli* host strains. For recombinant plasmid construction, the *Ph*ADH gene was amplified with primers containing NcoI and EcoRI restriction enzyme sites (forward 5′-TAGAATTCATGAGCAAGATGCGCGGTTTTC-3′, reverse 5′-ACCTCGAGTCACTCCTCTATGATGACC-3′). Digestion of the DNA was performed in the recommended buffer for 2–4 h using 10–15 U of the endonucleases (1–1.5 μL; 10 U/μL) for 0.5–1.5 μg DNA at 37 °C; followed by a ligation reaction at 3:1 molar ratio of insert to vector T4 DNA Ligase. The ligation mixture was incubated at 16–20 °C overnight and the mixture was used for transformation. In the plasmid transformation, 10 μL of the ligation product was added to 100 μL corresponding competent cells and the mixture was heat-shocked at 42 °C for 60 s. The selection of bacterial cells that carry the recombinant plasmids was then performed with antibiotic stress. *E. coli* BL21 (DE3) strains used in this research are sensitive to common antibiotics including kanamycin, while pET-30a has been engineered to harbor the genes for antibiotic resistance. The bacterial transformations were plated onto media containing 50 mg/mL kanamycin and only bacteria possessing the plasmid DNA would have the ability to form colonies. Under control of the T7-lac promoter, the recombinant *Ph*ADH was obtained in the cytoplasmic space when isopropyl β-D-1-thiogalactopyranoside (IPTG) was added to the 2YT medium with 1 mM ferrous. To optimize the growth condition, both the concentration of the inducer as well as the growth phase of recombinant cells at which it was added were tested. The inducer IPTG was added as a gradient of 0, 0.2 mM, 0.4 mM, 0.8 mM, and 1 mM; the optimum concentration of the inducer was detected by the amount of recombinant protein on the SDS- PAGE. Then, IPTG was added in the exponential phase when OD_600_ of the cell culture reached 0.4–1.0, which was recommended by the pET manual. However, induction at OD_600_ of 0.8 provided an ideal yield with high activity. Therefore, induction in this study was set at OD_600_ of 0.8.

### 2.4. Purification of Recombinant PhADH

All purification steps were carried out anaerobically using a fast-performance liquid chromatography (FPLC) system (Baie D’Urfe, QC, Canada) considering the oxygen sensitivity of native *Ph*ADH [17]. Since the enzyme was thermostable, a step of heat precipitation was applied prior to the column chromatography. The cell extract was incubated at 60 °C for half an hour, and the solution turned gel-like. The denatured proteins and cell debris in the cell crude extract were removed by centrifugation at 10,000× *g* for 30 min at room temperature. The supernatant containing enzyme activity were collected and pooled to a DEAE-Sepharose column (2.6 × 10 cm) equilibrated with buffer A (50 mM Tris-HCl with 2 mM dithiothreitol (DTT) and 2 mM sodium dithionite (SDT), pH 7.8). A linear gradient (0–0.5 M sodium chloride in buffer A) was applied at a flow rate of 2.5 mL/min and the *Ph*ADH was eluted and collected anaerobically.

### 2.5. Enzyme Assay of PhADH

Activity of *Ph*ADH was measured using the same method described by [17]. The optimal pH values of both native and recombinant *Ph*ADHs were determined by enzyme assay of butanol oxidation or butyraldehyde reduction. Standard enzyme assays at 80 °C for alcohol oxidation were carried out using a set of 100 mM buffers [17]: Tris/HCl (pH 8.0, 8.5, 9.0) and 3-(cyclohexylamino)-1-propanesulfonic acid (CAPS, pH 9.0, 9.7, 10.0, 10.5, 11.0, 11.5, and 12.0). The optimal pH value of the acetaldehyde-dependent reduction of native and recombinant *Ph*ADH was measured as between pH 6.0 to 9.0 using the following 100 mM buffers: 1,4-piperazine-bis-(ethanesulfonic acid) (PIPES, pH 6.0, 6.5, and 7.0), 4-(2-hydroxyethy)-1-piperazineethanesulfonic acid (HEPES, pH 7.0, 7.5, and 8.0), and Tris/HCl (pH 8.0, 8.5, 9.0). Moreover, the effect of the temperature on the enzyme activity was examined at temperatures from 30 to 95 °C and enzyme thermostability was evaluated by incubating the enzyme in sealed serum bottles at 70 °C and 90 °C, respectively. The residual activities of each sample at different time intervals were measured parallelly using the standard assay conditions. The effect of oxygen on enzyme activity was investigated by exposing the enzyme samples to air at room temperature and determining the residual activity after oxygen exposure. The exposure was performed in the presence and absence of 2 mM DTT and SDT. The residual activities of each sample at different time intervals were measured using the standard enzyme activity assay described above. One unit of *Ph*ADH activity is defined as the oxidation or formation of 1 µmol of NADPH per min under the assay conditions.

### 2.6. Other Methods

Protein concentration was determined by using the Bradford assay [30]. The purity of the enzyme preparation was determined using sodium dodecyl sulfate-polyacrylamide gel electrophoresis (SDS-PSAGE). After purification of the recombinant *Ph*ADH from *E. coli*, size exclusion chromatography was used in order to determine the molecular mass of its native form. The enzyme sample was loaded onto the gel filtration column Superdex 200 (2.6 × 60 cm) equilibrated in 50 mM Tris-HCl (pH 7.8) containing 100 mM KCl at a flow rate of 2 mL/min. The size of the native form of *Ph*ADH was calculated based on the elution volume of standard proteins (Pharmacia, NJ, USA) that contained blue dextran (molecular mass, Da, 2,000,000), thyroglobulin (669,000), ferritin (440,000), catalase (232,000), aldolase (158,000), bovine serum albumin (67,000), ovalbumin (43,000), chymotrysinogen A (25,000) and ribonuclease A (13,700).

## 3. Results and Discussion

### 3.1. Cloning and Sequencing of the Gene Encoding PhADH

**Figure 1:**
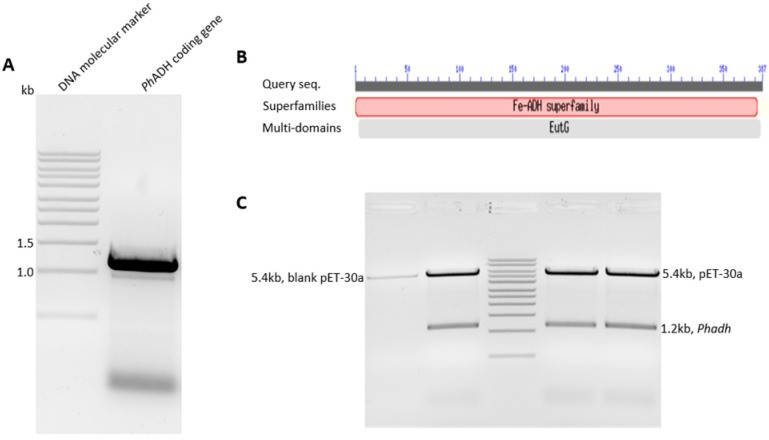
*Ph*ADH gene PCR amplification and its insertion into the plasmid pET-30: (**A**) PCR amplification of entire *Ph*ADH coding gene, indicated by a single DNA band between 1–1.5 kb on the agarose gel electrophoresis; (**B**) putative conserved domains of *P. hypogea* ADH. Fe-ADH superfamily, a group of uncharacterized iron-containing alcohol dehydrogenase, and EutG, an alcohol dehydrogenase; and (**C**) confirmation of the recombinant vector carrying the *Ph*ADH by enzyme digestion. The arrows point to the recombinant vector and blank pET-30a vector after enzyme digestion by Nco I and Eco RI. The 1.2 kb bands were released from recombinant vectors after enzyme digestion.

**Figure 2:**
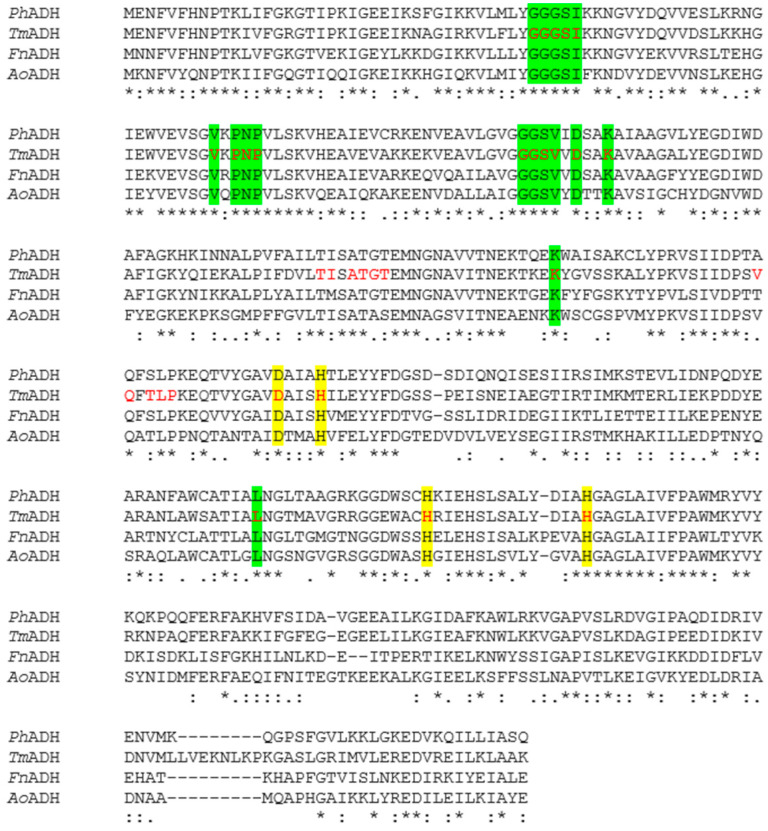
Amino acid sequence alignment among *Ph*ADH and its homologous enzymes. Sequence alignments with homologues in iron-containing ADH families and phylogenetic trees were constructed by the Clustal W tool with default parameters [31]. Yellow, putative catalytic iron-binding site. Green, putative motif of coenzyme NADP binding site. Red, residues in which the role has been shown in the crystal structure of TM0920 [6]. *Tm*ADH, *T. matritima* ADH (AAD35902); *Fn*ADH, *Fervidobacterium nodosum* ADH (ABS61516); *Ao*ADH, *Alkaliphilus orremlandii* ADH (ABW18251). ‘‘*’’, identical residues; ‘‘:’’, conserved substitutions; ‘‘.’’, semi-conserved substitutions; “-”, no corresponding residues.

Using the designed primers and PCR amplifications, DNA fragments with approximately 150 bp were obtained and sequenced; these were shown to be highly similar to an iron-containing ADH from *T. maritima* whose genome sequence is available. A series of PCRs were run by using primers designed based on both the conserved sequence analysis and newly obtained parts of the gene sequence. The final PCR product showed a specific 1.2 kb band on a 1% agarose gel, which was extracted and confirmed by sequencing (Figure 1A). Ultimately, an entire gene with a 1164 bp nucleotide sequence was obtained, encoding the 387 amino acids sequence with a calculated molecular weight of 43 kDa (GenBank: AMT84600.1) (Figure 2).

### 3.2. Sequence Analysis of PhADH

**Figure 3:**
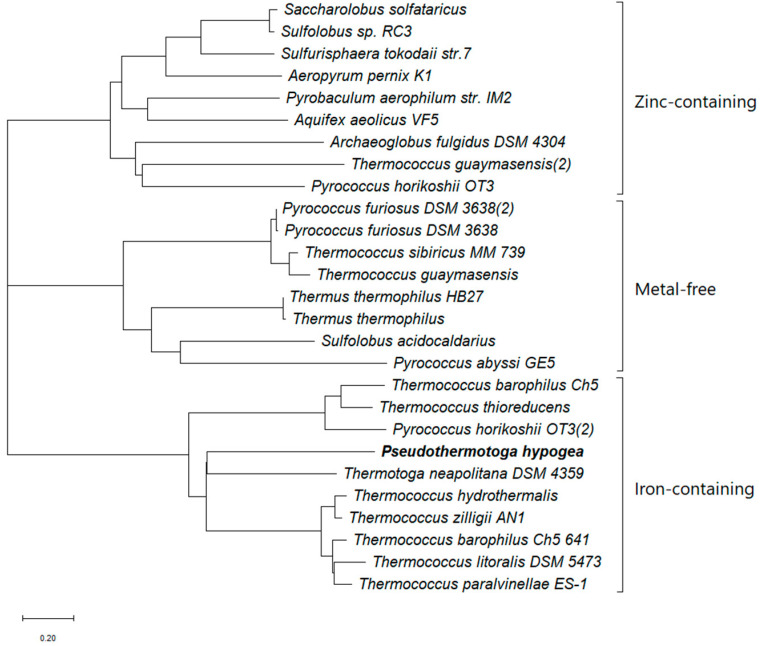
Phylogenetic comparison of the three types of alcohol dehydrogenases from hyperthermophiles. The sequences were aligned using CLUSTALW. MEGA 11 software was used to construct the tree, using the neighbour-joining tree method. The accession numbers of the sequences obtained from the NCBI database are: *Aeropyrum pernix* K1, BAA81251.2; *Aquifex aeolicus* VF5, AAC07327.1; *Archaeoglobus fulgidus* DSM 4304, AAB89145.1; *Pseudothermotoga hypogea*, AMT84600.1; *Pyrobaculum aerophilum* str. IM2, AAL64366.1; *Pyrococcus abyssi* GE5, CAB49186.1; *Pyrococcus furiosus* DSM 3638, AAL80198.1; *Pyrococcus furiosus DSM* 3638, AAC25556.1; *Pyrococcus horikoshii* OT3, BAA29746.1; *Pyrococcus horikoshii* OT3, BAA29834.1; *Saccharolobus solfataricus*, CAA09258.1; *Sulfolobus acidocaldarius*, WP_011278925.1; *Sulfolobus* sp. RC3, CAA87591.1; *Sulfurisphaera tokodaii* str.7, BAK54135.1; *Thermococcus barophilus* Ch5, ALM74514.1; *Thermococcus barophilus* Ch5 641, ALM74601.1; *Thermococcus guaymasensis*, WP_062370843.1; *Thermococcus guaymasensis*, ADV18977.1; *Thermococcus hydrothermalis*, CAA74334.1; *Thermococcus litoralis* DSM 5473, EHR78112.2; *Thermococcus paralvinellae* ES-1, ACK56133.1; *Thermococcus sibiricus* MM 739, ACS89385.1; *Thermococcus thioreducens*, ASJ12775.1; *Thermococcus zilligii* AN1, AAB63011.1; *Thermotoga neapolitana* DSM 4359, ACM22756.1; *Thermus thermophilus*, BBL81612.1; *Thermus thermophilus* HB27, WP_011172461.1.

The BLAST analysis of the deduced amino acid sequence showed a sequence identity of up to 72% to the iron-containing enzymes in the bacteria domain, especially *Thermotogales* such as *T. maritima* [32], *Thermotoga petrophila* [33]; a 60% identity to *Fervidobacterium nodosum* Rt17-B1 [34]; a 58% identity to *Thermosipho melanesiensis* [35]; a moderately high identity (46–58%) to enzymes in other thermophilic bacteria such as *Symbiobacterium thermophilum* [36] and *Thermoanaerobacter ethanolicus* X514 [37]. The *Ph*ADH also presented similarity to the mesophiles, e.g., a 54% identity to *Alkaliphilus oremlandii* ADH [38]. *Ph*ADH belonged to the group of iron-containing ADHs, which was verified by conserved motif searches, indicating that the enzyme belonged to a family of uncharacterized oxidoreductases of the iron-containing alcohol dehydrogenase (Figure 1B and Figure 2). The amino acid sequence alignment of *Ph*ADH and its homologous enzymes indicated that the co-factor binding and putative active site for binding iron were conserved (Figure 2). The sequence analysis provided further evidence that the *Ph*ADH was NADP-dependent and iron-containing, which matched the results of the biochemical characterization [17]. This is the first *Ph*ADH purified from this new type of hyperthermophilic bacterial ADHs. It showed closer relatedness to the bacterial iron-containing ADH from *Thermotoga neapolitana* (Figure 3), which is reported to be a bifunctional iron-containing enzyme [18]. The homologues of *P. hypogea* ADH seem to be abundant in bacteria but not in archaea, which could be an indication of the divergence of iron-containing ADHs in hyperthermophiles.

The primary structural analyses verified that the enzyme and its thermophilic homolog, *Fervidobacterium nodosum* alcohol dehydrogenase, had a higher ratio (molar fraction, >0.8% increase or decrease) for Ala, Arg, Lys, Thr and Val but a lower ratio for Asn, Glu, Tyr and Met compared to the ADH from the mesophile *Alkaliphilus oremlandii*. Particularly, the amino acid composition of *Ph*ADH had a higher ratio for Ala, Pro, Trp and Val but a lower ratio for Glu, Lys and Thr than that of the ADH from the *F. nodosum*. Furthermore, the exchange of hydrophilic and large hydrophobic residues in the thermophilic and mesopilic counterparts for the small hydrophobic amino acids, Ala, Pro and Val, in *Ph*ADH may also contribute to the higher thermostability of the enzyme by locking the enzyme in a conformation with a higher density of packing and decreased structural flexibility [39].

### 3.3. Modeling of the 3D Structure of the PhADH

**Figure 4:**
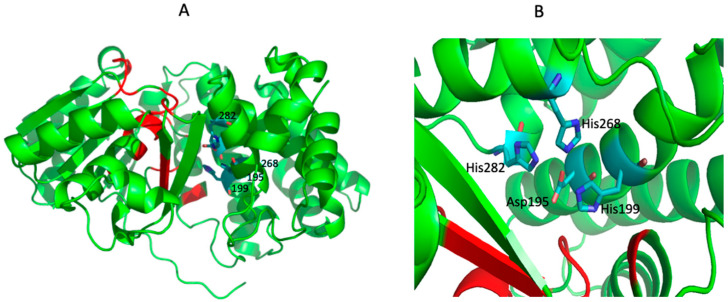
Predicted tertiary structure of *Ph*ADH monomer and the putative iron-binding site. The structure modeling was run on the Swiss Model server (https://swissmodel.expasy.org) using an iron-containing ADH from *T. maritima* (TM0820; PDB number: 1vljB) as the template: (**A**) residues in red are the putative NADP+-binding site, residues in blue are the putative iron-binding site; and (**B**) the vertical view of the putative iron-binding site, Asp195His199His268His282.

The *Ph*ADH amino-terminus contained a GGGS motif (residues 39–42) which is well recognized as being involved in the interactions of the pyrophosphate groups of NADP^+^ [6]. The predicted 3-D structure of *Ph*ADH was modulated by Pymol software (1.0rev1). The tertiary structural modeling of monomer of *Ph*ADH showed two typical domains, separating with a deep cleft (Figure 4). Like the 3-D structure of iron-containing ADH from *T. maritima*, the N-terminal domain was formed by an α/β region containing the dinucleotide-binding fold, whereas the C-terminal part was an all-helical domain responsible for the iron-binding. The putative active site motif was identified to be Asp195His199His268His282, and the NADP^+^-binding site was predicted to be G183XG185XXG188, respectively (Figure 4).

### 3.4. Overexpression and Purification of the Recombinant PhADH

**Figure 5:**
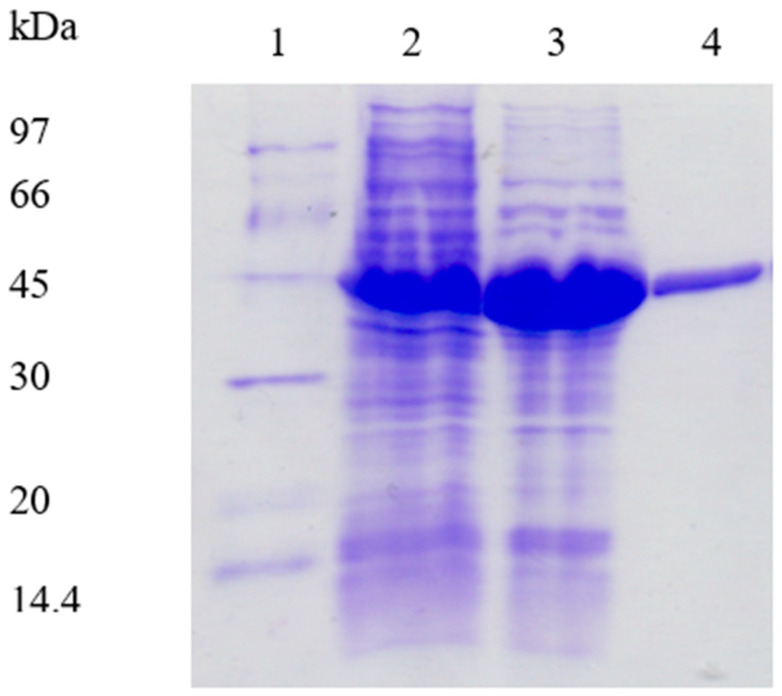
SDS-PAGE (10%) analysis of the purified recombinant *Ph*ADH. Lane 1, molecular weight marker; Lane 2, cell crude extract; Lane 3, cell crude extract heat treated for 30 min; Lane 4, purified recombinant *Ph*ADH.

*E. coli* was selected as a heterologous expression host in this research. However, the gene encoding *Ph*ADH has a different codon usage compared to the *E. coli*, which was analyzed by a graphical codon usage analyzer and showed a mean difference of 39.98%. To overcome a possible poor yield caused by codon bias, confirmed by sequencing, the isolated recombinant plasmids carrying the *Ph*ADH gene were transformed into *E. coli* BL 21-Rossetta expression strains, containing the extra plasmid for rarely used tRNAs codons AGA/AGG/AUA/CUA/GGA/CCC/CGG to rescue the poor expression by codon bias mainly caused by the rare tRNA in *E. coli*: AGG/AGA for arginine, AUA for isoleucine, and CUC for leucine. From 10% SDS-PAGE, a large amount of recombinant enzymes of around 43 kDa was produced in the presence of IPTG as an inducer (Figure 5).

Considering the thermostability of *Ph*ADH, the recombinant enzyme was purified from *E. coli* using a simplified procedure. Heat treatment was applied to the cell extract prior to liquid chromatography. Heating for 30 min at 60 °C caused nearly no loss of enzyme activity but significantly reduced the protein concentration. Subsequently, the recombinant *Ph*ADH was purified to homogeneity after DEAE-Sepharose anion exchange chromatography. The purified recombinant *Ph*ADH had a specific activity of 69 ± 3 U/mg compared to 68 ± 2 U/mg of the native enzyme [17] and presented a higher yield of 49%, which was higher than the 31% of the native enzyme purification (Table 1). An aliquot of the purified recombinant enzyme was loaded to the gel-filtration to determine the native molecular weight of the enzyme. The target enzyme was eluted at 192 mL, and the size of the recombinant enzyme was calculated to be approximately 84 ± 5 kDa. The SDS-PAGE analyses showed that the recombinant *Ph*ADH had a subunit size of 43 ± 2 kDa, suggesting that both native and recombinant enzymes were homodimers in their native forms.

The over-expression of *Ph*ADH was successful in the mesophilic host *E. coli* by pET vector, which is often selected for the expression of proteins from thermophiles. The recombinant enzyme was cytoplasmic and soluble, and no inclusion body was formed. The activity of recombinant *Ph*ADH from *E. coli* did not require heat activation. However, heat activation was essential to obtain maximally active forms of the iron-containing ADH from archaeal *T. hydrothermalis* [13] and 2,3-butanediol ADH from *P. furiosus* [40]. The activity of the recombinant *T. hydrothermalis* ADH increased 10–25% after 1 min incubation at 80 °C, while *P. furiosus* ADH was inactive without heat treatment and the highest activity was obtained after a 10 min incubation at 100 °C. Recombinant *T. paralvinellae* ES-1 ADH activity was unaffected by heat treating the cell-free extract [11], similar to what was observed with the recombinant short-chain ADH from *P. furiosus* [41].

### 3.5. Comparison of Properties between the Recombinant and Native PhADHs

**Figure 6:**
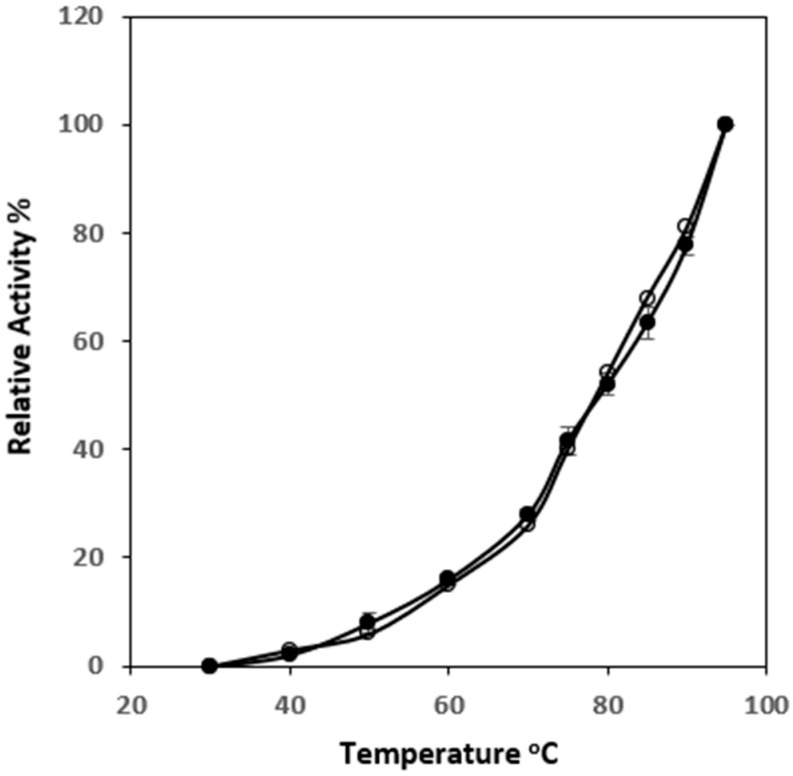
Temperature dependency of the purified *Ph*ADH. Filled circles, recombinant *Ph*ADH; open circles, native *Ph*ADH [42]. Enzyme activities were measured in the standard assay conditions, except that assay temperatures varied from 30 to 95 °C. The 100% relative activity was the highest activity value, which was 133 ± 5 U/mg at 95 °C.

Biochemical properties, including temperature dependency, optimum pH, thermostability and oxygen sensitivity of the recombinant enzyme, were extensively examined after purification and compared to the published corresponding features of its native form from *P. hypogea* [17]. In general, both enzymes had very similar catalytic properties. Over a temperature range from 30 to 95 °C, the activity of recombinant *Ph*ADH increased along with the increase of assay temperature, showing the same trend as the native enzyme (Figure 6). When the thermostability was tested at 70 °C, the activity of the recombinant enzyme remained up to 50% after 5 h in the presence of 2 mM DTT, however, the half-life (t_1/2_) of the recombinant enzyme was detected to be about one hour at 90 °C in the absence and presence of DTT.

**Figure 7:**
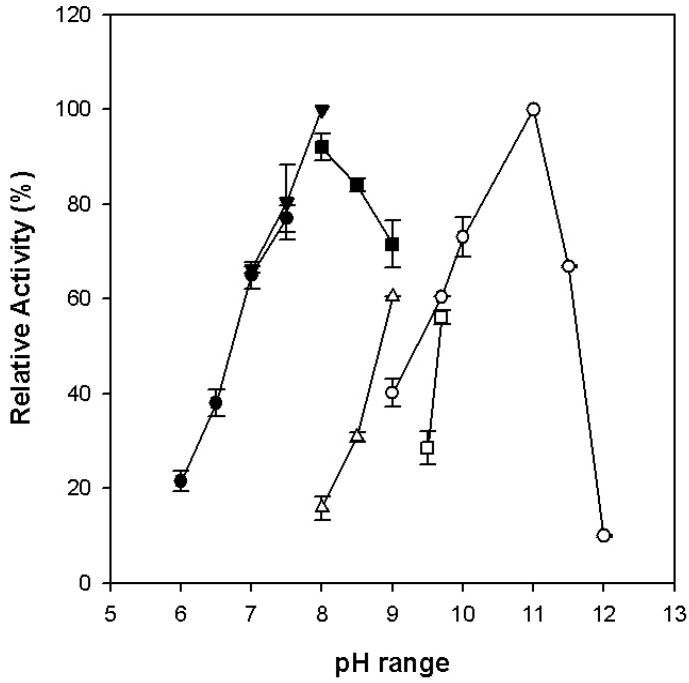
Optimum pH of the purified recombinant *Ph*ADH. Optimal pH values for alcohol oxidation and formation were determined by measuring the activities upon the oxidation of butanol (unfilled points) and the reduction in butyraldehyde (filled points), respectively. The buffers (100 mM) used were CAPS (unfilled circles), Tris/HCl (unfilled triangles and filled squares), glycine (unfilled squares), HEPES (filled converted triangles), and PIPES (filled circles).

**Figure 8:**
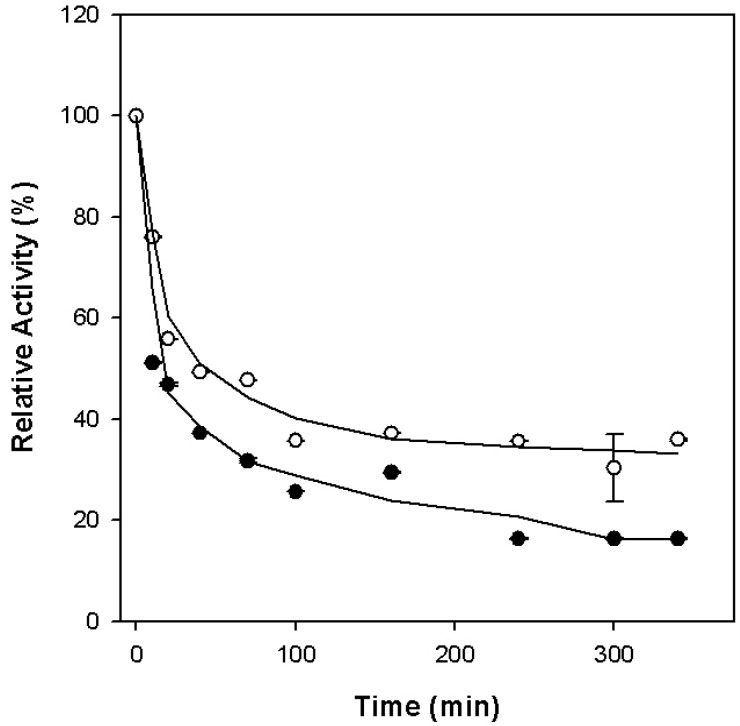
Oxygen sensitivity of the purified recombinant *Ph*ADH. Open circles, in the presence of 2 mM DTT and 2 mM SDT; filled circles, in the absence of 2 mM DTT and 2 mM SDT. The relative activity of 100% is equal to the ADH activity prior to exposure to air.

The effect of pH on enzyme activities was investigated with a set of 100 mM buffers ranging from pH 6.0 to 12.0. The optimal pH for alcohol oxidation was 11.0, while that for aldehyde reduction was 8.0 (Figure 7). Each enzyme activity worked optimally within a narrow pH range. When the buffer pH value was higher or lower than the optimum value, the activity of alcohol oxidation had a remarkable decrease. Both the native and recombinant enzymes were sensitive to oxygen. The residual activities of the recombinant enzyme reduced to 50% after exposure to the air at room temperature for about 20 and 50 min in the absence and presence of the reducing agents, sodium dithionite and dithiothreitol (Figure 8). While the native enzyme had lost about 80% of its activity in the first 30 min [17], it took about 4 h for the recombinant enzyme to lose the same percentage of its activity in the absence of the reducing agents. The reducing agents slowed down the oxidation process more quickly in the native enzyme in the early minutes of exposure to oxygen than it did in the recombinant enzyme. Although both were oxygen-sensitive, the recombinant enzyme appeared to be slightly more oxygen-tolerant than the native enzyme.

The catalytic parameters of the recombinant *Ph*ADH were confirmed to be the same as those of the native one [17], indicating that this iron-containing ADH was successfully produced in *E. coli* and that the heterologous expression in *E. coli* could potentially be used for the large-scale production of this recombinant iron-containing hyperthermophilic ADH. Notably, however, oxygen sensitivity limited the application of *Ph*ADH, and the construction of a more oxygen-tolerant enzyme is the focus of further study. Since protein lability is caused by oxidation or loss of Fe^2+^, the presence of reducing agents lessens the rate of oxidation of Fe^2+^ to Fe^3+^, which has less affinity to the enzyme iron-binding site. Iron substitution by divalent metal ions that could not be further oxidized (e.g., Zn^2+^, Co^2+^ etc.) at the catalytic site may provide further mechanistic insights into the nature of oxygen sensitivity. Moreover, protein engineering is a powerful approach to alter the catalytic properties of enzymes. Site-directed mutagenesis and direct evolution are the most commonly used approaches for protein engineering. In addition, site-directed mutagenesis is also widely used for identifying the role of specific amino acids in catalysis. In general, the 3-D structural information of enzymes is critical to understand the structure–function relationships for enzymes. The successful production of recombinant enzymes provides a sufficient amount of active enzymes for a crystallography study that will shed light on the design of site-directed mutagenesis.

## 4. Conclusions

*P. hypogea* is an extremely thermophilic bacterium that grows on a wide range of carbohydrates and produces ethanol as a metabolic end product. It is catalyzed by an iron-containing alcohol dehydrogenase. The gene encoding *P. hypogea* ADH (*Ph*ADH) was successfully cloned, sequenced, and over-expressed in a mesophilic host, *E. coli*. The obtained gene sequence has a length of 1164 bp, and the deduced amino acid sequence consists of 387 amino acid residues. It showed high sequence identity to iron-containing ADHs from other *Thermotoga* species. Typical iron- and NADP-binding motifs were identified, respectively, as Asp195His199His268His282 and Gly39Gly40Gly41Ser42. Structural modeling showed that the N-terminal domain of *Ph*ADH contains a α/β-dinucleotide-binding motif and its C-terminal domain contains an α-helix-rich region including an iron-binding motif. The recombinant *Ph*ADH was soluble, active, and thermostable, with a subunit size of 43 ± 1 kDa, revealed by SDS-PAGE analyses. The activity of the purified recombinant *Ph*ADH was shown to have the same properties as the native enzyme. The successful expression of recombinant *Ph*ADH in *E. coli* significantly enhanced the yield of enzyme production and thus will facilitate further investigation of the catalytic mechanisms of iron- containing ADHs through X-ray crystallography analysis in the future. The potential industrial, pharmaceutical, and biotechnological applications of the recombinant enzyme can be exploited further.

## Figures and Tables

**Table 1:** Purification of the recombinant *Ph*ADH.

Steps	Total Protein (mg)	Total Activity (U)	Specific Activity (U/mg)	Purification Fold	Yield (%)
Cell-free extract	240	3552	14.8	1	100
Heat-treatment	62.8	2427.2	38.5	2.6	68
DEAE-Sepharose	25.1	1734.4	69.1	4.7	49

## Data Availability

Data are contained within the article.
